# The Immediate and Sustained Effects of Moderate-Intensity Continuous Exercise and High-Intensity Interval Exercise on Working Memory

**DOI:** 10.3389/fpsyg.2022.766679

**Published:** 2022-02-15

**Authors:** Hong Mou, Shudong Tian, Qun Fang, Fanghui Qiu

**Affiliations:** Department of Physical Education, Qingdao University, Qingdao, China

**Keywords:** acute exercise, cognitive function, working memory, 2-back task, time course

## Abstract

This study investigated the immediate and delayed effects of moderate-intensity continuous exercise (MICE) and high-intensity interval exercise (HIIE) on working memory. Fifty healthy young adults (mean age = 19.96 ± 1.03 years) engaged in (1) a MICE session, 20 min of continuous running on a treadmill at an intensity of 40–59% of heart rate reserve (HRR); (2) a HIIE session, 10 sets of 1 min running at an intensity of 90% HRR, interspersed by 1 min self-paced walking at 50% HRR; and (3) a control session, resting in a chair and reading books for 24 min. A spatial 2-back task was performed to assess working memory before, immediately after and 30 min after each intervention. Reaction time in the 2-back task was significantly reduced immediately after both MICE and HIIE interventions. The enhanced working memory associated with HIIE sustained for 30 min after the exercise, whereas the beneficial effects associated with MICE returned to the pre-exercise level at 30 min after the exercise. These results suggest that although both MICE and HIIE enhance working memory in young adults, the positive effect sustains longer in HIIE than that in MICE. The current study extends the existing knowledge base by suggesting that improvements in working memory with HIIE last longer than with MICE.

## Introduction

Working memory refers to the ability to actively store and manipulate task-relevant information in a short period of time, which is a core component of executive function (Baddeley, 2010; Diamond, 2013). Working memory is essential in achieving academic success and solving complex cognitive tasks in daily life (Kao et al., 2017a). Evidence has shown that acute exercise produces transient improvement in working memory (Hussey et al., 2020; Liu et al., 2020; Olivo et al., 2021). Previous studies used a single bout of moderate-intensity continuous exercise (MICE), a traditional exercise, as an intervention program to explore the relation between exercise and working memory. Significant improvement in working memory after MICE cessation has been reported (Weng et al., 2015; Chen et al., 2016; Dodwell et al., 2019). In addition to traditional continuous exercise, high-intensity interval exercise (HIIE), which is defined as repetitive high-intensity exercise interspersed with recovery periods of light intensity (Kao et al., 2017b), has been recognized as one of the most effective methods for improving exercise capacity, metabolism, and cardiorespiratory fitness (Weston et al., 2014; Batacan et al., 2017; Dolci et al., 2020). Recently, HIIE has been widely investigated for its positive influence on working memory during the period following the cessation of the exercise bout (Ai et al., 2021; Hsieh et al., 2021).

Although the benefits of acute exercise on working memory is well known, it may vary according to exercise modality (Chen et al., 2020). Kao et al. (2021) used a modified Sternberg task to assess working memory in healthy young adults and found that only HIIE enhanced the processing speed during memory retrieval compared with MICE and control condition. Northey et al. (2019) also found that the HIIE intervention elicited positive moderate-to-large effects on working memory in comparison with MICE. However, some studies proposed that MICE maximizes exercise-induced cognitive benefits versus HIIE (McMorris and Hale, 2012; Pyke et al., 2020) and contributes to a significant improvement in working memory performance relative to pre-intervention (Tsai et al., 2021). The existing literature remains inconsistent about which modality of exercise, MICE or HIIE, is the more effective in improving working memory. Thus, further research to establish the relationship between exercise modality and working memory is necessary.

Another concern which needs to be addressed in the subsequent research is the time point of working memory assessment after exercise (Pontifex et al., 2019). Most previous studies exploring the effects of acute exercise on working memory focused on the immediate effects (up to 5 min after exercise) of acute exercise (Chen et al., 2014; Soga et al., 2015; Bediz et al., 2016), with only a few studies examined sustained effects after exercise (Pontifex et al., 2009; Tsujii et al., 2013; Kamijo and Abe, 2019). A meta-analysis investigating the effects of acute exercise on cognitive function (including working memory) has indicated that the effects of exercise on cognition vary with the time of performing cognitive tasks (Chang et al., 2012). The cognitive tests displayed the greatest effects 1–15 min after exercise, and then, these effects faded following a longer (>15 min) delay. Cooper et al. (2018) used HIIE as an intervention and observed changes in working memory performance immediately after and 45 min after the intervention and only found an increase in performance at the maximum level of working memory load in the Sternberg paradigm (working memory load in the current study has three levels) immediately after exercise. In contrast, two studies (with the MICE and HIIE interventions, respectively) reported that the improvement in working memory capacity was prominent at the end of the exercise intervention and continuously elevated up to 30 min (Pontifex et al., 2009; Martinez-Diaz et al., 2020). However, these results contradict Chang et al. (2012) who suggested that physiological responses to exercise (e.g., heart rate, brain-derived neurotrophic factor (BDNF), endorphins, serotonin, and dopamine) predicted effects on executive function performance. When executive function was tested immediately after exercise, lower intensity exercise produced appropriate levels of physiological mechanisms that resulted in more beneficial effects on executive function; however, higher intensity exercise produced the strongest effects when executive function was tested delayed after exercise. To our knowledge, there are no studies that have directly compared the sustainable effects of MICE and HIIE on working memory, and thus, the effects of different exercise protocol on lasting improvements in working memory should be examined.

The purpose of this study is to investigate the different effects of acute MICE versus HIIE on working memory and whether this effect remains stable after a 30 min recovery period using duration-matched exercise protocols in healthy young adults. Given that drive theories suggested a linear relationship between intensity of exercise and executive function such that the benefit scales in relation to increasing intensity (Chang et al., 2012), we hypothesized that (1) both the MICE and HIIE conditions would improve working memory compared with the control condition and (2) HIIE would have a longer duration of positive effect on working memory than MICE.

## Materials and Methods

### Participants

Fifty healthy young adults (20 females) aged 18–22 years (mean age = 19.96 ± 1.03 years) were recruited from Qingdao University to participate in this study. Participants completed the Physical Activity Readiness Questionnaire (PAR-Q; Thomas et al., 1992) to exclude potential exercise risks. All eligible participants were right-handed, free of any reported neurological, cardiovascular, or pulmonary diseases and had normal or corrected-to-normal vision. The sample size is determined based on *a priori* power (G^*^power 3.1.9.2) analysis with a small-to-moderate effect size (*η*^2^ = 0.23; Etnier et al., 2016), *α* level of 0.05 and a power (1—*β*) of 0.80 at the group level, resulting in at least a sample size of 33 to detect similar significant effects. Considering potential attrition of participants during the experiment, we recruited a total of 50 participants. Each participant received cash compensation after the experiment. The study was approved by the Medical Ethics Committee of the Affiliated Hospital of Qingdao University and all participants signed informed consent. Demographic characteristics data have been shown in Table 1.

**Table 1:** Demographic characteristics data (*M ± SD*).

Variables	Mean ± SD	Range
Sample size (n)	50	
Gender (male/female)	30/20	
Age (years)	19.96 ± 1.03	18–22
Height (cm)	173.38 ± 8.59	153–190
Weight (kg)	65.51 ± 14.03	39–98
BMI (kg.m^−2^)	21.59 ± 3.29	16.33–30.71
HR_max_ (bpm)	193.46 ± 6.16	180–210
RHR (bmp)	69.18 ± 8.27	55–90
HRR (bmp)	124.28 ± 9.65	104–153
Mean MICE HR (bpm)	135.00 ± 4.94	100–158
Mean HIIE HR (bpm)	163.83 ± 7.77	108–194
MICE RPE	12.22 ± 2.12	9–17
HIIE RPE	16.10 ± 2.43	11–20

*BMI, body mass index; HR_max_, maximum heart rate; RHR, resting heart rate; HRR, heart rate reserve; HR, heart rate; MICE, moderate-intensity continuous exercise; HIIE, high-intensity interval exercise; and RPE, ratings of perceived exertion*.

### Spatial 2-Back Task

**Figure 1:**
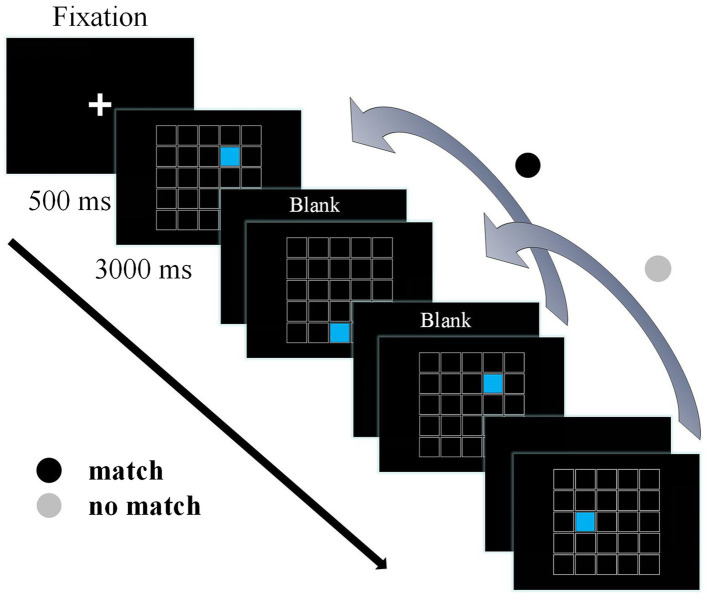
The spatial 2-back task. A fixation point was presented before stimulus onset, followed by a 500 ms stimulus and a 3,000 ms reaction time, for a total stimulus time window of 3,500 ms.

A modified version of a spatial 2-back task was programmed using E-Prime version 2.0 (Psychology Software Tools Inc., Pittsburgh, PA, United States) to assess working memory (Drollette et al., 2012). The display screen consisted of 25 white-framed 1.5 × 1.5 cm small black squares combined into a large square. A small square filled with blue was presented randomly in one of the 25 squares for 500 ms, followed by a 3,000 ms of response interval in which a black screen was displayed, resulting in a total response window of 3,500 ms (Figure 1). Participants were required to respond as quickly and accurately as possible to press the F button on the keyboard when the position of the current stimulus matched the position of blue square presented in the previous two trials (i.e., match trials), and press the J button on the keyboard when the two trials were not matched (i.e., no match trials). It was considered a false alarm (FA) when a participant incorrectly identified a trial with no match when the F button was pressed. The task contains two blocks of 23 trials each block, including seven match trials, 14 no match trials, and two beginning trials to which do not need to respond. There were 23 practice trials before the formal experimental trials. Behavioral performance for the 2-back task included mean accuracy, mean reaction time (RT), false alarm rate, and *d’* scores (*d’*; Target discrimination performance parameters). Calculation of *d’* scores used the formula provided by Sorkin (Sorkin, 1999), z (adjusted target accuracy)—z (adjusted false alarm rate). Larger values of *d’* indicate better ability to discriminate targets from non-targets (Scudder et al., 2016).

### Experimental Procedure

**Figure 2:**
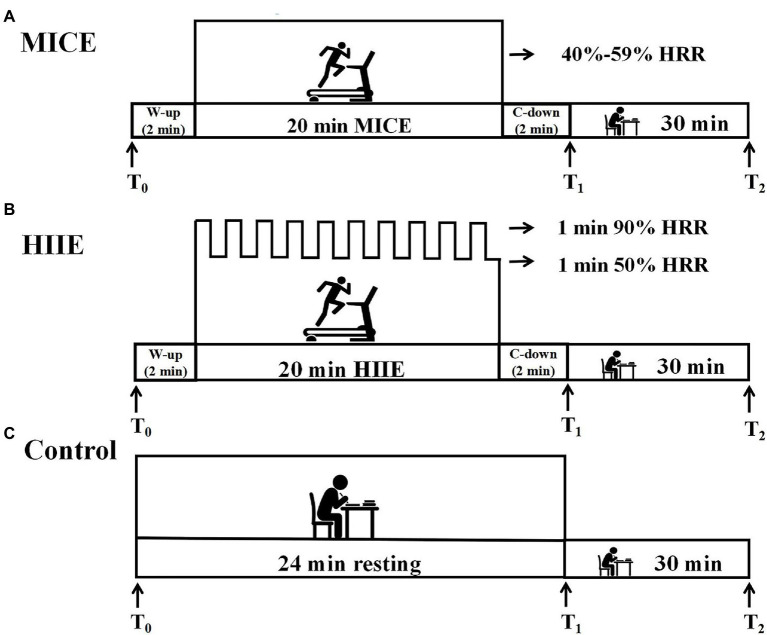
Schematic of the study design. Working memory was assessed pre-intervention (T_0_) and at two time points after intervention, including immediately (T_1_) and 30 min (T_2_). MICE: moderate-intensity continuous exercise. HIIE: high-intensity interval exercise. W-up: warm-up. C-down: cool-down. Sections **(A–C)** of the figure represent the intervention protocols for the HIIE, MICE and control group respectively.

The protocol adopted a within-subjects design. All participants engaged in four sessions. The interval between each two sessions was ≥7 days to minimize any effects of the previous session, and for a given participant, the four sessions were completed at approximately the same time of the day. Participants were instructed to avoid strenuous physical activity, caffeinated beverages, or alcoholic drinks at least 24 h before each session. During the first session, participants completed relevant questionnaires and performed a graded exercise test (GXT) using a treadmill (ICON 705CST) for estimating maximum heart rate (HR_max_) (Ferguson, 2014). The initial speed of the test was 8.5 km/h with the grade of 3%; then, the speed of the treadmill was increased by 0.5 km/h every 1 min and the grade was kept constant until the participant became volitional exhausted. A Polar H10 heart rate strap (Polar, Kemple, Finland) measured HR throughout the test and assessed the rating of perceived exertion (RPE) immediately after the test (Borg, 1970). HR_max_ was determined when participants met three of the following four criteria: (a) a plateau in heart rate resulting in no longer rising with an increase in the speed of the treadmill, (b) a peak HR ≥ age-predicted HR_max_ [208 – (0.7^*^age)] (Mahon et al., 2010), (c) RPE ≥ 17, and (d) subjective volitional exhaustion was reported. During the other three sessions, participants completed one of the three interventions (i.e., MICE, HIIE, and control) in a randomized counterbalanced order and were instructed to perform a spatial 2-back task test before the intervention (T_0_), immediately after (T_1_), and 30 min after the intervention (T_2_). The entire protocol took participants approximately 6 h. The Polar H10 heart rate strap was used to monitor real-time heart rate throughout the exercise intervention. Figure 2 shows the experimental procedure. Participants were asked to keep quiet and to refrain from doing anything unrelated to the experimental intervention.

### Intervention Protocols

All exercise interventions were completed on a treadmill. We tailored the intensity of each exercise intervention to the participants based on their estimated heart rate reserve (HRR) based on their HR_max_ minus their resting heart rate (RHR). The target HR during exercise was calculated from the following formula: target HR = (% exercise intensity ^*^ HRR) + RHR. During the MICE session, participants completed a 2 min warm-up, followed by 20 min of continuous running on a treadmill an intensity of 40–59% HRR, and a 2 min cool-down (Garber et al., 2011). During the HIIE session, participants completed a 2 min warm-up, followed by a 20 min interval exercise, and a 2 min cool-down. During the interval exercise, participants repeated 10 bouts of 1 min running at an intensity of 90% HRR, separated by 1 min of self-paced walking at 50% HRR (Andrews et al., 2020). The Borg scale was completed immediately after the two exercise interventions to assess the participants’ RPE (Borg, 1970). During the control protocol, participants were instructed to rest on a chair and read books that were unrelated to exercise in a quiet room for 24 min which matched the duration of the exercise intervention (2 min warm-up, 20 min exercise, and 2 min cool-down). The intervention protocols are illustrated in Figure 2.

### Statistical Analysis

Statistical analyses were conducted using the Statistical Package for Social Sciences software (SPSS version 25.0, Chicago, United States), with a significant level set at *p* = 0.05. In RT analysis, trials with RT below 300 ms or above two standard deviations (*SD*) were discarded. The total amount of trials discarded by these trimmers was less than 3% of the original dataset. Accuracies and correct RTs of the 2-back task were analyzed using a two-way repeated-measures analysis of variance (ANOVA), with condition (MICE, HIIE, and control) and time point (T_0_, T_1_, and T_2_) as within-subject factors. False alarm rate and *d’* scores were examined using a 3 (condition: MICE, HIIE, control) × 3 (time: T_0_, T_1_, T_2_) repeated-measures ANOVA. Paired-samples *t*-tests were used for HR and RPE analyses in MICE and HIIE conditions. For ANOVA that showed significant main or interaction effects among different factors, paired *t*-tests with Bonferroni corrections were used for *post-hoc* comparisons. Partial eta-squared was used to indicate the effect sizes.

## Results

### Acute Exercise Performance

A paired-sample *t*-test of mean HR and RPE for MICE and HIIE found that HIIE elicited a higher HR (163.83 ± 7.77 bpm vs. 135.00 ± 4.94 bpm, *p* < 0.001) and greater PRE (16.10 ± 2.43 vs. 12.22 ± 2.12, *p* < 0.001) relative to MICE (Table 1).

### Accuracy of the 2-Back Task

**Figure 3:**
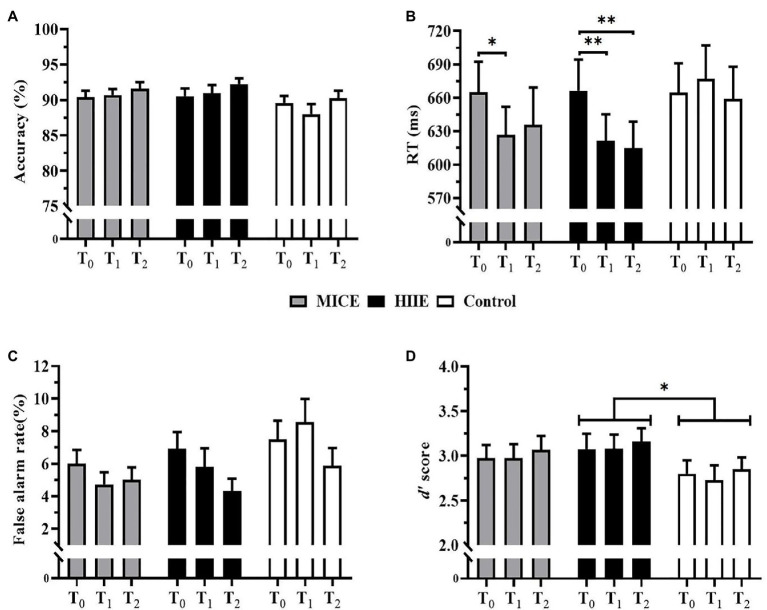
Mean accuracy **(A)**, mean reaction time (RT) **(B)**, false alarm rate **(C)**, and *d*’ scores **(D)** for 2-back task. Error bars depict standard errors. MICE: moderate-intensity continuous exercise. HIIE: high-intensity interval exercise. ^*^p < 0.05 and ^**^p < 0.01.

The two-way repeated-measures ANOVA on accuracy showed that no significant interaction of time point × condition [*F* (4, 196) = 0.67, *p* = 0.617, *η*^2^ = 0.01] or main effect of condition [*F* (2, 98) = 2.90, *p* = 0.060, *η*^2^ = 0.06] was observed. A significant main effect of time point was confirmed [*F* (2, 98) = 3.57, *p* = 0.032*, η*^2^ = 0.07]. *Post-hoc* analysis showed that the accuracy was significantly higher 30 min after intervention (T_2_) compared to that immediately after the intervention (T_1_; *p* = 0.033), while there was no significant difference in either T_1_ or T_2_ relative to T_0_ (*p*s ≥ 0.131; see Figure 3A).

### Reaction Time of the 2-Back Task

The two-way repeated-measures ANOVA on RT revealed a significant interaction of time point × condition [*F* (4, 196) = 2.83, *p* = 0.042, *η*^2^ = 0.06]. *Post-hoc* tests showed that RT was significantly faster in the immediate after intervention (T_1_) for both HIIE (*p* = 0.002) and MICE (*p* = 0.013) than pre-intervention (T_0_). However, the benefit of HIIE on RT was maintained for 30 min after the intervention (T_2_; *p* = 0.003), while RT before the MICE intervention was not significantly different from that 30 min after the intervention (T_2_; *p* = 0.235). *Post-hoc* test also revealed that RT at the T_1_ time point was significantly shorter in the HIIE condition than that in the control condition (*p* = 0.013). In the control condition, no significant differences were observed among the pre-intervention (T_0_), immediate after intervention (T_1_) and 30 min after intervention time points (T_2_; *p*s ≥ 0.284).

There was a significant main effect of time point [*F* (2, 98) = 8.92, *p* < 0.001, *η*^2^ = 0.17]. The *post-hoc* test showed that the RT was significantly slower before the intervention (T_0_) compared with those immediately (T_1_; *p* < 0.001) and 30 min (T_2_; *p* = 0.001) after the intervention, while RTs were not significantly different between T_1_ and T_2_ time points (*p* > 0.9). There was no significant main effect of the condition [*F* (2, 98) = 2.00, *p* = 0.141, *η*^2^ = 0.04; see Figure 3B].

### False Alarm Rate and *d*’ Scores

Analysis of the false alarm rate showed that there was no significant interaction effect of time point × condition [*F* (4, 196) = 1.74, *p* = 0.157, *η*^2^ = 0.03] or significant main effect of condition [*F* (2, 98) = 3.04, *p* = 0.061, *η*^2^ = 0.06]. A significant main effect of time point was identified [*F* (2, 98) = 5.45, *p* = 0.006, *η*^2^ = 0.10]. *Post-hoc* analysis showed that the false alarm rate was significantly lower 30 min after the intervention (T_2_) compared to that before the intervention (T_0_; *p* = 0.006). No significant differences were detected in the false alarm rate between T_0_ and T_1_ (*p* > 0.9) or T_1_ and T_2_ (*p* = 0.065; see Figure 3C).

For *d’* score, there was no significant interaction of time point × condition [*F* (4, 196) = 0.04, *p* = 0.998, *η*^2^ < 0.01] or main effect of time point [*F* (2, 98) = 0.51, *p* = 0.601, *η*^2^ = 0.01]. A significant main effect of condition was observed [*F* (2, 98) = 3.35, *p* = 0.039, *η*^2^ = 0.06]. *Post-hoc* analysis revealed that the HIIE condition had higher *d’* scores compared to the control condition (*p* = 0.024). However, there were no significant differences in *d’* scores between MICE and control condition (*p* = 0.336) and between HIIE and MICE (*p* > 0.9; see Figure 3D).

## Discussion

The purpose of this study was to examine the immediate and delayed effects of acute MICE and HIIE relative to a resting control condition on working memory in healthy young adults using a spatial 2-back task. Results indicated that both MICE and HIIE have a beneficial effect on working memory in healthy young adults, but there were differences in the duration of the improvement between the two exercises. Improvements in working memory immediately after exercise were similar in the HIIE and MICE protocols. However, the benefit after HIIE lasted for 30 min of post-exercise recovery, during which MICE returned to the pre-exercise level. As we hypothesized, HIIE produced longer working memory improvement than MICE.

Consistent with previous studies (Chen et al., 2016; Dodwell et al., 2019), the current study found that MICE improved working memory immediately after the intervention, as evidenced by an overall decrease in RT with no difference in response accuracy for the 2-back task relative to that before the acute exercise. This finding may suggest that MICE improved the processing efficiency of working memory processes (McMorris et al., 2011). However, the MICE-induced improvement in working memory returned to pre-exercise levels 30 min after exercise. This is inconsistent with previous studies reporting that improvement in working memory induced by MICE (30 min of motor driven treadmill exercise) was not only reflected immediately after exercise, but also remained 30 min after exercise. Ji et al. (2017) also found a significant reduction in RT for working memory tasks after moderate-intensity exercise at 60% HRR (30 min treadmill running), and this improvement was maintained until 30 min after exercise. The discrepancies between these studies and our findings may stem from differences in the duration of the exercise intervention (Chang et al., 2015). A meta-analysis found that performance on cognitive tests was facilitated when participants completed an exercise longer than 20 min (Lambourne and Tomporowski, 2010). Improvement in working memory was not observed 30 min after MICE in the present study, probably because our exercise intervention was not long enough to produce a significant effect.

The present study demonstrated that working memory improved immediately after HIIE and lasted up to 30 min. This finding supports some recent studies reporting that cognitive improvements can be maintained for a period of time after HIIE. For example, Martinez-Diaz et al. (2020) used the Digit Span Test (DST) to assess working memory in healthy young men and found that DST scores obtained 30 min after exercise remained higher than those assessed before the exercise. Williams et al. (2020) used high-intensity intermittent games-based activity to explore the moderating effect of physical fitness on the acute exercise-cognition relationship and measured working memory performance using the Sternberg paradigm. Results revealed that working memory improved in the high fitness group 45 min after exercise. Our findings validate previous studies reporting that HIIE improves working memory (Moreau and Chou, 2019; Ai et al., 2021), confirming that HIIE-induced improvements in working memory can be sustained until at least 30 min after exercise.

Previous studies have demonstrated that HIIE was a more effective strategy for improving inhibitory control and cognitive flexibility compared to MICE (Tian et al., 2021a,b). In the present study, we provide evidence that HIIE is also effective in improving working memory. The improvement in working memory after the MICE and HIIE interventions implied some predictions related to describing the neurobiological mechanisms underlying the effects of exercise on cognitive function. As a stressor, the transient effects of acute exercise on working memory are commonly explained by energetic arousal. This arousal is mainly expressed as increases in neural activation or general physiological arousal measured by heart rate, RPE, or other biological indices (Lambourne and Tomporowski, 2010; Netz et al., 2016). Studies have shown that arousal levels increase with exercise intensity (Yerkes and Dodson, 1908), with cognitive performance rising to an optimal level along with increased exercise-induced arousal levels (Lambourne and Tomporowski, 2010). The RPE was significantly greater after HIIE compared to MICE, which may lead to a higher level of arousal in the organism with HIIE than MICE, resulting in a longer duration of improvement in working memory. Changes in BDNF accompanying exercise participation can also contribute to improved working memory (Aaron et al., 2015). Previous studies have found acute increases in BDNF concentrations in human blood serum after a single bout of physical exercise (Knaepen et al., 2010), and the elevated levels are greater after high-intensity exercise than after low-intensity exercise (Ferris et al., 2007). Acute exercise-induced changes in BDNF concentrations can predict memory-related cognitive performance (Skriver et al., 2014), possibly explaining the longer duration of HIIE on working memory than MICE. Further, the increased cerebral blood flow promoted by exercise may also be a possible mechanism for the improvement in working memory after acute exercise. Acute exercise promotes increased cerebral blood flow (Ellis et al., 2017; Steventon et al., 2021), which facilitates the transport of nutrients and oxygen to the brain (Vogiatzis et al., 2011), and the resulting increased oxygenation benefits broader cognition, particularly in the frontal areas of the brain that support executive functions (Lambrick et al., 2016). Some studies using functional near-infrared spectroscopy have demonstrated increased cortical activation in the prefrontal cortex after exercise, corresponding to an increase in cognitive performance (Byun et al., 2014). Meanwhile, since interval exercise requires more complex coordination of motor movements and higher demands on executive functions, it may result in a greater increase in cerebral blood flow to the prefrontal cortex compared to continuous exercise and has a greater impact on working memory (Lambrick et al., 2016).

A few limitations must be taken into consideration. Firstly, the study compared the effects of two different modalities of exercise on working memory, HIIE, and MICE, but the energy expenditure induced by exercise was not equal, so it was difficult to determine whether the differential effects of HIIE and MICE on working memory were related to energy expenditure. Secondly, we propose that exercise-induced energy arousal increased BDNF concentrations and cerebral blood flow might play important roles in improving working memory. However, we did not perform direct measurements of these physiological indices. Further studies are needed to measure exercise-induced changes in biological indicators, such as BDNF concentration and cerebral blood flow. Finally, the current study found that the benefits of HIIE on working memory lasted up to 30 min after exercise cessation. However, since the working memory test ends at that point, it is unclear whether the benefits of HIIE on working memory can be sustained for much longer.

## Conclusion

The present study indicated the acute effects of treadmill-based HIIE and MICE on the sustainability of working memory in healthy young adults. Specifically, the MICE intervention significantly facilitated working memory only immediately after exercise. However, the enhanced working memory associated with the HIIE intervention was not only observed immediately after exercise, but also lasted for at least 30 min after exercise. Thus, the differential effects of HIIE and MICE on working memory provide support for the importance of exercise modality as a possible modulator of the relationship between acute exercise and working memory. The finding that HIIE is a more time-efficient method to improving cognitive performance compared to MICE may have some social significance. Long-term sedentary study and work with intervals of short-duration HIIE intervention could generate better learning and work performance that relies on working memory.

## Data Availability Statement

The raw data supporting the conclusions of this article will be made available by the authors, without undue reservation.

## Ethics Statement

The studies involving human participants were reviewed and approved by Affiliated Hospital of Qingdao University. The patients/participants provided their written informed consent to participate in this study.

## Author Contributions

HM, ST, and FQ contributed to conception and design of the study and provided the datasets. HM carried out the analysis. HM, ST, QF, and FQ wrote the initial draft. All authors contributed to manuscript revision, read, and approved the submitted version.

## Funding

The present study was funded by the Foundation for Science Research Famous Achievement Award in Higher Institution (Humanities and Social Sciences, RZ2100004646).

## Conflict of Interest

The authors declare that the research was conducted in the absence of any commercial or financial relationships that could be construed as a potential conflict of interest.

## Publisher’s Note

All claims expressed in this article are solely those of the authors and do not necessarily represent those of their affiliated organizations, or those of the publisher, the editors and the reviewers. Any product that may be evaluated in this article, or claim that may be made by its manufacturer, is not guaranteed or endorsed by the publisher.
